# The effect of human mobility and control measures on the COVID-19 epidemic in China

**DOI:** 10.1126/science.abb4218

**Published:** 2020-03-25

**Authors:** Moritz U. G. Kraemer, Chia-Hung Yang, Bernardo Gutierrez, Chieh-Hsi Wu, Brennan Klein, David M. Pigott, Open COVID-19 Data Working Group, Louis du Plessis, Nuno R. Faria, Ruoran Li, William P. Hanage, John S. Brownstein, Maylis Layan, Alessandro Vespignani, Huaiyu Tian, Christopher Dye, Oliver G. Pybus, Samuel V. Scarpino

**Affiliations:** 1 Department of Zoology, University of Oxford, Oxford, UK,; 2 Harvard Medical School, Harvard University, Boston, MA, USA.; 3 Boston Children’s Hospital, Boston, MA, USA.; 4 Network Science Institute, Northeastern University, Boston, MA, USA.; 5 School of Biological and Environmental Sciences, Universidad San Francisco de Quito USFQ, Quito, Ecuador.; 6 Mathematical Sciences, University of Southampton, Southampton, UK.; 7 Institute for Health Metrics and Evaluation, Department of Health Metrics, University of Washington, Seattle, WA, USA.; 8 Harvard T.H. Chan School of Public Health, Boston, MA, USA.; 9 Mathematical Modelling of Infectious Diseases Unit, Institut Pasteur, UMR2000, CNRS, Paris, France.; 10 Sorbonne Universite, Paris, France.; 11 ISI Foundation, Turin, Italy.; 12 State Key Laboratory of Remote Sensing Science, College of Global Change and Earth System Science, Beijing Normal University, Beijing, China.; 13 Department of Pathobiology and Population Sciences, The Royal Veterinary College, London, UK.

## Abstract

The ongoing COVID-19 outbreak expanded rapidly throughout China. Major behavioral, clinical, and state interventions have been undertaken to mitigate the epidemic and prevent the persistence of the virus in human populations in China and worldwide. It remains unclear how these unprecedented interventions, including travel restrictions, affected COVID-19 spread in China. We use real-time mobility data from Wuhan and detailed case data including travel history to elucidate the role of case importation on transmission in cities across China and ascertain the impact of control measures. Early on, the spatial distribution of COVID-19 cases in China was explained well by human mobility data. Following the implementation of control measures, this correlation dropped and growth rates became negative in most locations, although shifts in the demographics of reported cases were still indicative of local chains of transmission outside Wuhan. This study shows that the drastic control measures implemented in China substantially mitigated the spread of COVID-19.

The outbreak of COVID-19 spread rapidly from its origin in Wuhan, Hubei Province, China (*1*). A range of interventions have been implemented following the detection in late December 2019 of a cluster of pneumonia cases of unknown etiology, and identification of the causative virus SARS-CoV-2 in early January 2020 (*2*). Interventions include improved rates of diagnostic testing, clinical management, rapid isolation of suspected cases, confirmed cases and contacts and, most notably, restrictions on mobility (hereafter called cordon sanitaire) imposed on Wuhan city on 23^rd^ January. Travel restrictions were subsequently imposed on 14 other cities across Hubei Province and partial movement restrictions were enacted in many cities across China. Initial analysis suggests that the Wuhan cordon sanitaire resulted in an average delay of COVID-19 spread to other cities of 3 days (*3*), but the full extent of the effect of the mobility restrictions and other types of interventions on transmission has not been examined quantitatively (*4*–*6*). Questions remain over how these interventions affected the spread of SARS-CoV-2 to locations outside of Wuhan. We here use real-time mobility data, crowdsourced line-list data of cases with reported travel history, and timelines of reporting changes to identify early shifts in the epidemiological dynamics of the COVID-19 epidemic in China, from an epidemic driven by frequent importations to local transmission.

## Human mobility predicts the spread and size of epidemics in China

**Fig. 1:**
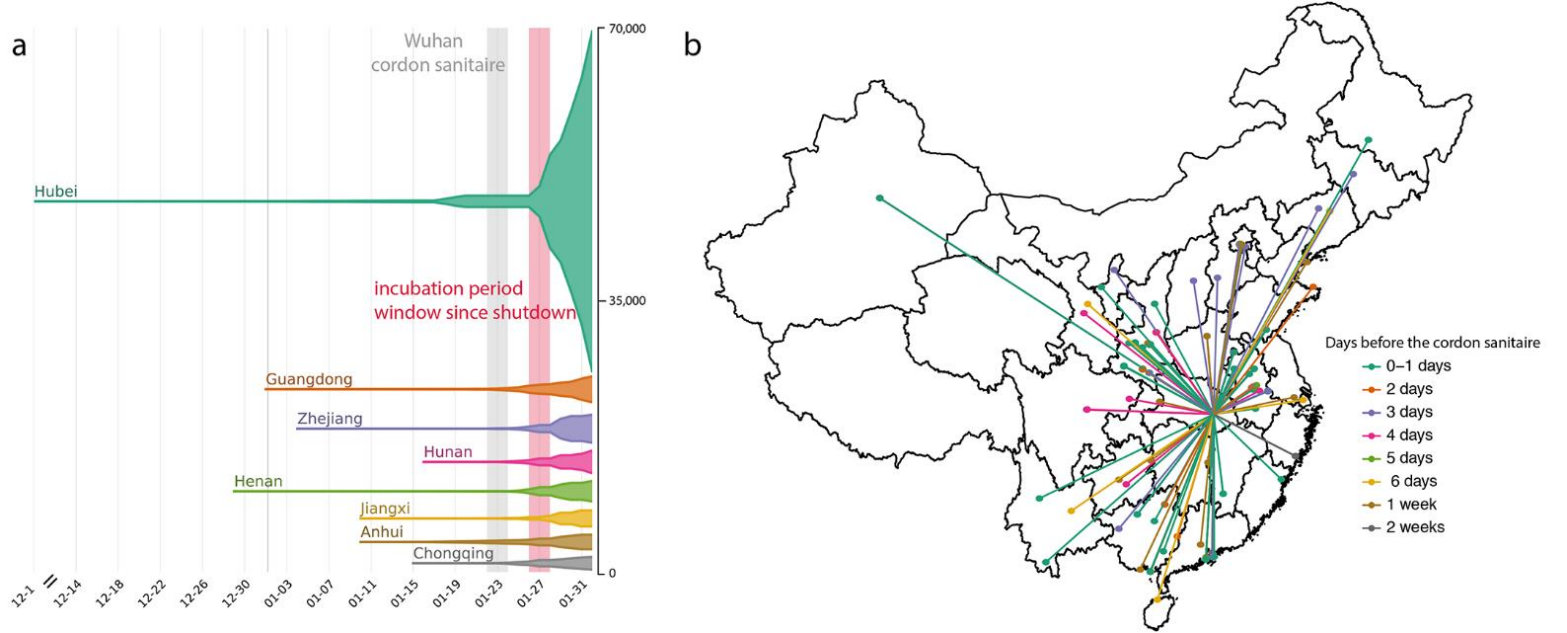
Number of cases and key dates during the epidemic. (**a**) The epidemic curve of the COVID-19 outbreak in provinces in China. Vertical lines and boxes indicate key dates such as implementation of the cordon sanitaire of Wuhan (grey) and the end of the first incubation period after the travel restrictions (red). The thin grey line represents the closure of Wuhan seafood market on 1^st^ January 2020. The width of each horizontal tube represents the number of reported cases in that province. (**b**) Map of COVID-19 confirmed cases (n = 554) that had reported travel history from Wuhan before travel restrictions were implemented on January 23, 2020. Colors of the arrows indicate date of travel relative to the date of travel restrictions.

As of 1^st^ March 2020, 79,986 cases of COVID-19 were confirmed in China (Fig. 1a) (*7*). Reports of cases in China were mostly restricted to Hubei until 23^rd^ January 2020 (81% of all cases), after which most provinces reported rapid increases in cases (Fig. 1a). We built a line list dataset from reported cases in China with information on travel history and demographic characteristics (*8*). We note that the majority of early cases (before 23^rd^ January 2020, Materials and Methods) reported outside of Wuhan had known travel history to Wuhan (57%) and were distributed across China (Fig. 1b), highlighting the importance of Wuhan as a major source of early cases. However, initial testing was focused mainly on travelers from Wuhan, potentially biasing estimates of travel related infections upwards (Materials and Methods). Among cases known to have traveled from Wuhan before 23^rd^ January 2020, the time from symptom onset to confirmation was 6.5 days (SD: 4.2; fig. S2), providing opportunity for onward transmission at the destination. More active surveillance reduced this interval to 4.8 days (SD: 3.03; fig. S2) for those who travelled after 23^rd^ January 2020.

To identify accurately a timeframe for evaluating early shifts in SARS-CoV-2 transmission in China, we first estimated from case data the average incubation period of COVID-19 infection (*i.e.* the duration between time of infection and symptom onset (*9*, *10*)). Since infection events are typically not directly observed, we estimate incubation period from the span of exposure during which infection likely occurred. Using detailed information on 38 cases for whom both the dates of entry to and exit from Wuhan are known, we estimate the mean incubation period to be 5.1 days (std. dev. = 3.0 days; fig. S1), similar to previous estimates from other data (*11*, *12*). In subsequent analyses we add an upper estimate of one incubation period (mean + 1 standard deviation = 8 days) to the date of Wuhan shutdown, in order to delineate the date before which cases recorded in other provinces might represent infections acquired in Hubei (i.e., 1^st^ February 2020; Fig. 1a).

In order to understand whether the volume of travel within China could predict the epidemic outside of Wuhan, we analyzed real-time human mobility data from Baidu Inc., together with epidemiological data from each province (Materials and Methods). We investigated spatio-temporal disease spread to elucidate the relative contribution of Wuhan to transmission elsewhere and evaluate how the cordon sanitaire may have impacted it.

**Fig. 2:**
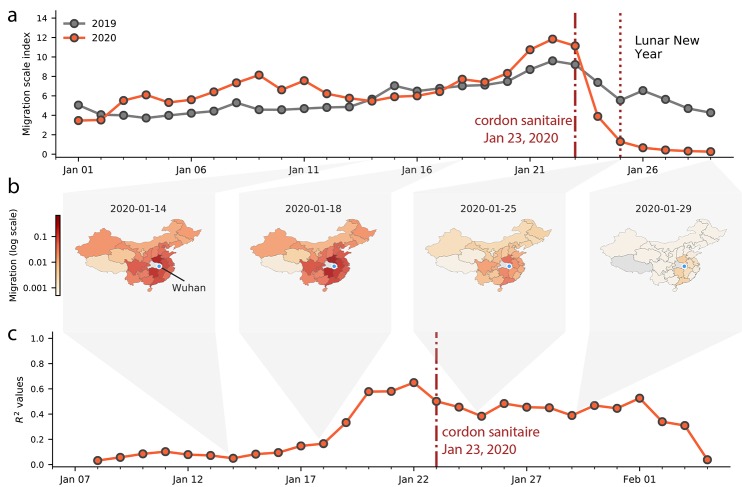
Human mobility, spread and synchrony of COVID-19 outbreak in China. (**a**) Human mobility data extracted in real time from Baidu. Travel restrictions from Wuhan and large scale control measures started on January 23,2020. Dark and red lines represent fluxes of human movements for 2019 and 2020, respectively. (**b**) Relative movements from Wuhan to other provinces in China. (**c**) Timeline of the correlation between daily incidence in Wuhan and incidence in all other provinces, weighted by human mobility.

Among cases reported outside Hubei province in our dataset, we observe 515 cases with known travel history to Wuhan and a symptom onset date before 31^st^ January 2020, compared with only 39 after 31^st^ January, 2020, illustrating the effect of travel restrictions (Figs. 1b and 2a and fig. S3). We confirm the expected decline of importation with real-time human mobility data from Baidu Inc. Movements of individuals out of Wuhan increased in the days before the Lunar New Year and the establishment of the cordon sanitaire, before rapidly decreasing to almost no movement (Fig. 2, a and b). The travel ban appears to have prevented travel in and out of Wuhan around the time of the Lunar New Year celebration (Fig. 2a) and likely reduced further dissemination of SARS-CoV-2 from Wuhan.

To test the contribution of the epidemic in Wuhan to seeding epidemics elsewhere in China we build a naïve COVID-19 GLM (*13*) model of daily case counts (Materials and Methods). We estimate the epidemic doubling time outside Hubei to be 4.0 days (range across provinces of 3.6 - 5.0 days) and estimate the epidemic doubling time within Hubei to be 7.2 days, consistent with previous reports (*5*, *12*, *14*, *15*). Our model predicts daily case counts across all provinces with relatively high accuracy (as measured with a pseudo-R^2^ from a negative binomial GLM) throughout early February 2020, and when accounting for human mobility (Fig. 2c and tables S1 and S2), consistent with an exploratory analysis (*6*).

We find that the magnitude of the early epidemic (total number of cases until February 10, 2020) outside of Wuhan is remarkably well predicted by the volume of human movement out of Wuhan alone (R^2^ = 0.89 from a log-linear regression using cumulative cases; fig. S8). Therefore cases exported from Wuhan prior to the cordon sanitaire appear to have contributed to initiating local chains of transmission, both in neighboring provinces (e.g., Henan) and in more distant provinces, (e.g., Guangdong and Zhejiang; Figs. 1a and 2b). Further, the frequency of introductions from Wuhan are also predictive of the size of the early epidemic in other provinces (controlling for population size) and thus the probability of large outbreaks (fig. S8).

After 1^st^ February 2020 (corresponding to one mean + one SD incubation period after the cordon sanitaire and other interventions were implemented), the correlation of daily case counts and human mobility from Wuhan decreased (Fig. 2c), indicating that variability among locations in daily case counts was better explained by factors unrelated to human mobility, such as local public health response. This suggests that while travel restrictions may have reduced the flow of case importations from Wuhan, other local mitigation strategies aimed at halting local transmission increased in importance later.

**Fig. 3:**
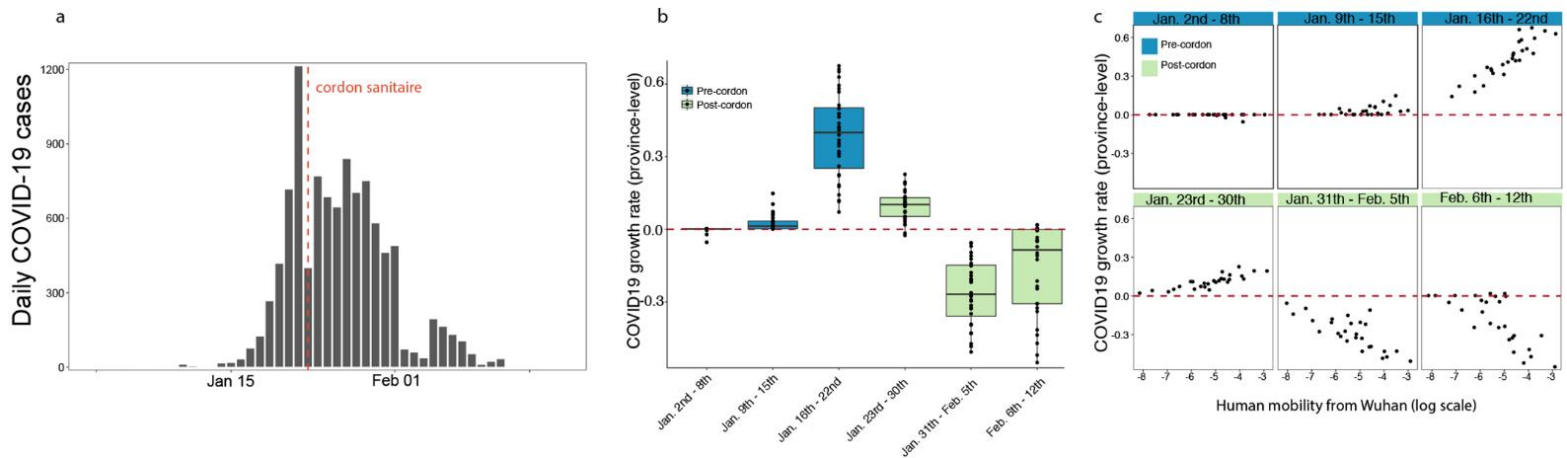
Human mobility explains early epidemic growth rate in China. (**a**) Daily counts of cases in China. (**b**) Time series of province-level growth rates of the COVID-19 epidemic in China. Estimates of the growth rate were obtained by performing a time-series analysis using mixed-effect model of lagged, log linear daily case counts in each province (**Materials and Methods**). Above the red line are positive growth rates and below are growth rates are negative rates. Blue indicates dates before the implementation of the cordon sanitaire and green after. (**c**) Relationship between growth rate and human mobility at different times of the epidemic. Blue indicates before the implementation of the cordon sanitaire and green after.

We estimate also the growth rates of the epidemic in all other provinces (Materials and Methods). Interestingly, we find that all provinces outside Hubei experienced faster growth rates between January 9^th^ – January 22^nd^, 2020 (Fig. 3, a and b, and fig. S4b) which was the time before travel restrictions and substantial control measures were implemented (Fig. 3c and fig. S6); this is also apparent from the case counts by province (fig. S6). In the same period, variation in the growth rates are almost entirely explained by human movements from Wuhan (Fig. 3c and fig. S9), consistent with theory of infectious disease spread in highly coupled metapopulations (*16*, *17*). Following the implementation of drastic control measures across the country, growth rates become negative (Fig. 3b), indicating that transmission was successfully mitigated. The correlation of growth rates and human mobility from Wuhan becomes negative, i.e., provinces with larger mobility from Wuhan prior to the cordon sanitaire (but also larger number of cases overall) have more rapidly declining growth rates of daily case counts. This could be due partly to travel restrictions but also to the fact that control measures may have been more drastic in locations with larger outbreaks driven by local transmission (see more detail in section “Current role of imported cases in Chinese provinces”).

The travel ban coincides with increased testing capacity across provinces in China. An alternative hypothesis is that the observed epidemiological patterns outside Wuhan are the result of increased testing capacity. We test this hypothesis by including differences in testing capacity before and after the rollout of large-scale testing in China on 20^th^ of January 2020 (the date that COVID-19 became a Class B notifiable disease (*18*, *19*)) and test the impact of this binary variable on the predictability of daily cases (Materials and Methods). We plot the relative improvement in the prediction of our model (based on normalized residual error) of (i) a model that includes daily mobility from Wuhan and (ii) a model that includes testing availability (see more details in Materials and Methods). Overall, the inclusion of mobility data from Wuhan produces a significant improvement in the model’s prediction (delta-BIC > 250, (*20*)) over a naive model that considers only autochthonous transmission with a doubling time of 2-8 days (Fig. 3b). Of the 27 provinces in China reporting cases through February 6^th^, 2020, we find that in 12 provinces the largest improvements in prediction can be achieved using mobility only (fig. S5). In 10 provinces, both testing and mobility improve the model’s prediction, and in only one province (Hunan) is testing the most important factor improving model prediction (fig. S5). We conclude that laboratory testing during the early phase of the epidemic is critical, however, mobility out of Wuhan remains the main driver of spread prior to the cordon sanitaire. Large scale molecular and serological data will be important to investigate further the exact magnitude of the impact of human mobility compared to other factors.

## Current role of imported cases in Chinese provinces

Since case counts outside Wuhan have decreased (Fig. 3b), we can further investigate the current contribution of imported cases to local epidemics outside Wuhan by investigating case characteristics. Age and sex distributions can reflect heterogeneities in the risk of infection within affected populations. To investigate meaningful shifts in the epidemiology of the COVID-19 outbreak through time, we examined age and sex data for cases from different periods of the outbreak, and from individuals with and without travel from Wuhan. However, details of travel history exist for only a fraction of confirmed cases and this information is particularly scant for some provinces (e.g., Zhejiang and Guangdong). Consequently, we grouped confirmed cases into four categories: (*I*) early cases with travel history (early = reported before 1^st^ Feb), (*II*) early cases without travel history, (*III*) later cases with travel history (later = reported between 1^st^ – 10^th^ Feb), (*IV*) later cases without travel history.

**Fig. 4:**
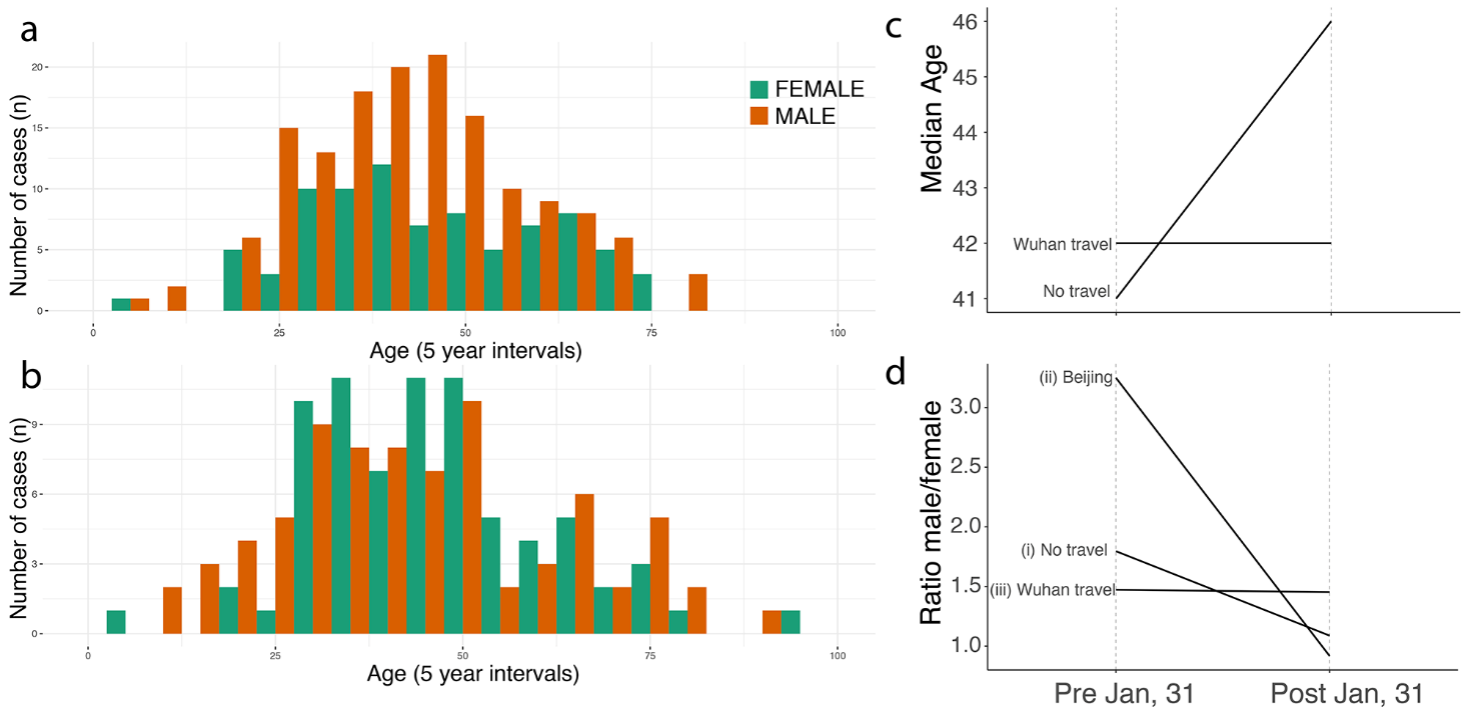
Shifting age and sex distributions through time. (**a**) Age and sex distributions of confirmed cases with known travel history to Wuhan. (**b**) Age and sex distributions of confirmed cases that had no travel history. (**c**) Median age for cases reported early (before 1^st^ Feb) and those reported later (between 1^st^ – 10^th^ February. Full distributions are shown in fig. S7. (**d**) Change through time in the sex ratio of (i) all reported cases in China with no reported travel history, (ii) cases reported in Beijing without travel history, and (iii) cases known to have travelled from Wuhan.

Using crowdsourced case data, we found that cases with travel history (categories *I* and *III*) had similar median ages and sex ratios in both the early and later phases of the outbreak (41 vs 42 years old, 50% interquartile interval: 32.75 vs 30.75 and 54.25 vs 53.5 respectively, p-value > 0.1; 1.47 vs. 1.45 males per female, respectively; Fig. 4d and fig. S7). Early cases with no information on travel history (category *II)* had a similar median age and sex ratio to those with known travel history (42 years old (50% interquartile interval: 30.5 – 49.5, p-value > 0.1) and 1.80 males per female; Fig. 4d). However, the sex ratio of later cases without reported travel history (category *IV*) shifted to approximately 1:1 (57 male vs. 62 female, X_2_ test, p-value < 0.01), as expected under a null hypothesis of equal transmission risk (Fig. 4, a, b, and d; see also (*21*, *22*) and the materials and methods) and the median age in this group increased to 46 (50% interquartile interval: 34.25 – 58, *t* test: p-value < 0.01) (Fig. 4 a, b, and c, and fig. S7). We hypothesize that many of the cases with no known travel history in the early phase were indeed travelers that contributed to disseminating SARS-CoV-2 outside of Wuhan. The shift toward more equal sex ratios and older ages in non-travellers after 31^st^ January 2020 confirm the finding that epidemics outside Wuhan were then driven by local transmission dynamics. The case definition changed to include cases without travel history to Wuhan after 23^th^ January 2020 (Materials and Methods).

## Discussion

Containment of respiratory infections is particularly difficult if they are characterized by relatively mild symptoms or transmission before the onset of disease (*23*, *24*). Intensive control measures, including travel restrictions, have been implemented to limit the spread of COVID-19 in China. Here, we show that travel restrictions are particularly useful in the early stage of an outbreak when it is confined to a certain area that acts as a major source. However, travel restrictions may be less effective once the outbreak is more widespread. The combination of interventions implemented in China were clearly successful in mitigating spread and reducing local transmission of COVID-19, although in this work it was not possible to definitively determine the impact of each intervention. Much further work is required to determine how to balance optimally the expected positive effect on public health with the negative impact on freedom of movement, the economy, and society at large.

## Supplementary Materials

science.sciencemag.org/cgi/content/full/science.abb4218/DC1 Materials and Methods Supplementary Text Figs. S1 to S9 Tables S1 and S2 List of members of the Open COVID-19 Data Working Group References (*26*–*40*)
