# Supplementary material for: The effect of human mobility and control measures on the COVID-19 epidemic in China
Source: Science. 2020 Mar 25:eabb4218. doi: 10.1126/science.abb4218 (PMC7146642; doi:10.1126/science.abb4218)
Supplement: Supplementary file 1 [file abb4218-Kraemer-SM.pdf]

science.sciencemag.org/cgi/content/full/science.abb4218/DC1

Supplementary Materials for

The effect of human mobility and control measures on the COVID-19 epidemic in China

Moritz U. G. Kraemer*, Chia-Hung Yang, Bernardo Gutierrez, Chieh-Hsi Wu, Brennan Klein, David M. Pigott, Open COVID-19 Data Working Group, Louis du Plessis, Nuno R. Faria, Ruoran Li, William P. Hanage, John S. Brownstein, Maylis Layan, Alessandro Vespignani, Huaiyu Tian, Christopher Dye, Oliver G. Pybus*, Samuel V. Scarpino*

*Corresponding author. Email: s.scarpino@northeastern.edu (S.V.S.); oliver.pybus@zoo.ox.ac.uk (O.G.P.); moritz.kraemer@zoo.ox.ac.uk (M.U.G.K.)

Published 25 March 2020 on *Science* First Release
DOI: 10.1126/science.abb4218

This PDF file includes:

Materials and Methods
Supplementary Text
Figs. S1 to S9
Tables S1 and S2
List of Members of the Open COVID-19 Data Working Group
References

Materials and Methods

Epidemiological data

No officially reported line list was available for cases in China (*26*). We use a standardised protocol (*27*) to extract individual level data from December 1st, 2019 - February 10th, 2020 (*8*). Sources are mainly official reports from provincial, municipal or national health governments. Data included basic demographics (age, sex), travel histories and key dates (dates of onset of symptoms, hospitalization, and confirmation). Data were entered by a team of data curators on a rolling basis and technical validation and geo-positioning protocols were applied continuously to ensure validity. A detailed description of the methodology is available (*8*). Lastly, total numbers were matched with officially reported data from China and other government reports. For sensitivity, GLM analyses (see below) were performed with case counts from the World Health Organization.

Proportions of symptomatic travelers

The proportion of cases who travelled while symptomatic was assessed from a subset of 236 cases for whom the dates of symptom onset and departure from Wuhan were available. Residency was split into three categories: Wuhan, China and International. Foreigners living in Wuhan were categorized as Wuhan and patients with missing Wuhan residency were either kept as missing values or categorized according to their country of origin. Both parametric (χ^2 test, (*28*)) and non-parametric (exact Fisher test, (*29*)) tests were performed and the uncertainty in proportions was assessed by the standard deviation of sample proportions.

Statistical inference of the incubation period

The incubation period is the time interval between infection and symptom onset. We assumed that cases travelling from Wuhan were exposed during their stay in Wuhan. We estimated the incubation period from 38 travelling cases returning from Wuhan with known dates of symptom onset, entry and exit. The end of the exposure period was assumed to be the exit travel date except if symptom onset occurred prior to the exit date (in which case exposure was assumed to have occurred prior to symptom onset). The start of the exposure period corresponded to the entry date. We assumed that the incubation period could not exceed 30 days.

For each case, the minimum and maximum incubation period was derived from the dates of entry, exit and symptom onset

$$IC_{max} = onset - entry$$

$$IC_{min} = onset - exit$$

We fitted a truncated gamma distribution (0 to 30 days) and estimated the mean and variance of the incubation period using Markov Chain Monte Carlo (MCMC) in a Bayesian framework using an uninformative prior distribution. We derived the likelihood as follows:

$$L = \frac{P_\Gamma(IC \leq IC_{max} + 1) - P_\Gamma(IC \leq IC_{min})}{P_\Gamma(IC \leq 30)}$$

A Metropolis-Hastings algorithm was implemented in R. Marginal posteriors were sampled from a chain of 5,000 steps after discarding a burn-in of 50 steps. Convergence was inspected visually.

Models of shifting age and sex distributions

Age and sex distributions are important in understanding risk of infection across populations. Assuming risk to be distributed relatively equally across a population, as an outbreak evolves age

and sex distributions should follow the underlying population structure. Varying degrees of immunity and exposure may shift these distributions (*30*). To examine whether the ongoing outbreak shifted from an epidemic concentrated in Wuhan and among travelers from Wuhan to an epidemic that was self-sustained in provinces across China we use age and sex data from different periods of the outbreak for individuals with reported travel history and no known travel history. We define two periods of the outbreak, an "early" phase, starting with the first reports in early December and ending a set number of days after the Wuhan shutdown. This was selected to be 8 days after the Wuhan shutdown, which conservatively corresponds to one incubation period + 1SD (see above) after the shutdown. After that date (i.e. 1st Feb 2020; the "later" phase) we assume that most reported transmissions in provinces outside of Wuhan are the result of local transmission. We further divided our data in those that had cases with known travel history to Wuhan and those who did not. Then we produce the following summary statistics:

1. Average age stratified by sex for all cases with reported travel history to Wuhan.
2. Average age stratified by sex for all cases with no reported travel history to Wuhan in the period between December 1, 2019 - January 31, 2020.

We then compare these with:

3. Average age stratified by sex for all cases with no reported travel history to Wuhan in the period between January 31, 2020 - February 10, 2020.

Model M1 compares the distribution of age and sex among travelers to the reported infections outside Wuhan with no known travel history. In case these two distributions are similar, import

driven epidemic can be concluded. Under our *model assumptions M2*, if the epidemic was driven largely by importations across the two time periods, all age and sex distributions should mirror those of the reported traveler infections. Under our *model assumptions M3*, if the epidemic was driven by other factors (i.e., local transmission), the two distributions should vary across the two time periods.

We cannot exclude the possibility that shifts in distributions may be due to heightened awareness among the general population which may have increased reporting in female cases later in the epidemic. Further, more work will be necessary to understand the differential risk of severe or symptomatic disease to fully understand the age and sex distributions in this outbreak. For example, why there are relatively few reports of cases <18y old. However, as for other respiratory pathogens symptomatic and severe infection were more concentrated in older populations. We do not intend to make any general statements about differential risk but were more interested in shifts in reported cases across multiple geographies in China.

Real time human mobility data

We extract human mobility data from the Baidu Qianxi web platform, which presents daily population travels between cities or provinces tracked through the Baidu Huiyan system. The data do not represent numbers of individual travelers but rather an index of relative movements constructed by Baidu's proprietary methods which are correlated with human mobility (*31*) (http://qianxi.baidu.com/). In particular, two pieces of information are collected. First, we extract a series of migration scale indices for traveling out of Wuhan, from January 1st to February 10, both in 2019 and 2020. Second, we obtain the proportion of human movement from Wuhan were

bound for each of 31 provinces in China. These proportions are available for January 1st - February 10, 2020. Based on this data we had access to both changes in mobility volume and changes in mobility direction. See more detailed descriptions of the human movement data here: (*32*, *33*). As of 2017, Baidu Inc's. mapping service had a 30% market share in China (*34*).

Review of interventions, testing capacity and reporting shifts

We reviewed the literature and online social media to understand the key timings of interventions and announcements that are relevant for disease transmission across China. We collated information about the type (e.g., announcement of outbreak, travel restrictions, isolation of patients, etc.), geographic location (e.g., city where available, province), and timing (specific date or date range).

COVID-19 case definitions:

Definitions of probable and confirmed COVID-19 cases have changed throughout the epidemic. We collected data from official sources describing the timing and specifics of the case definitions.

From January 18-22:

Probable: Need to satisfy (i) and (ii):

i. Clinical symptoms: (1) fever; (2) imaging showing pneumonia typical of the disease; (3) during early disease, total white cells normal or reduced, or lymph cell count reduced.

ii. Epidemiologic history: (1) within 2 weeks of symptom onset, Wuhan travel or resident history; or within 2 weeks of symptom onset, contact with persons from Wuhan who had fever with respiratory symptoms; or belong to a cluster.

Confirmed: Need to satisfy criteria for probable case and have a real-time quantitative polymerase chain reaction (RT-qPCR) positive result from sputum, nasopharyngeal swabs, lower respiratory tract secretions or other sample tissue, or genome sequencing highly similar with known SARS-CoV-2. available strains.

From January 22-23:

Probable: Need to satisfy (i) and any one epidemiologic history described in (ii):

i. Clinical symptoms: (1) fever; (2) imaging showing pneumonia typical of the disease; (3) during early disease, total white cells normal or reduced, or lymph cell count reduced

ii. Epidemiologic history: (1) within 2 weeks of symptom onset, Wuhan travel or resident history; (2) within 2 weeks of symptom onset, contact with persons from Wuhan who had fever with respiratory symptoms; (3) belong to a cluster or had epidemiologic link with confirmed cases.

Confirmed: Need to satisfy criteria for probable case and have a RT-qPCR positive result from respiratory or blood samples, or genome sequencing highly similar with known SARS-CoV-2. available strains.

From January 23-27:

Probable: Need to satisfy (i) and (ii):

i. Clinical symptoms: (1) fever; (2) imaging showing pneumonia typical of the disease; (3) during early disease, total white cells normal or reduced, or lymph cell count reduced

ii. Epidemiologic history: within 2 weeks of symptom onset, Wuhan travel or resident history; or within 2 weeks of symptom onset, contact with persons from Wuhan who had fever with respiratory symptoms, or belong to a cluster.

Confirmed: Need to satisfy criteria for probable case and have a RT-qPCR positive result from sputum, nasopharyngeal swabs, lower respiratory tract secretions, or other samples, or genome sequencing highly similar with known SARS-CoV-2. available strains.

From January 27-February 5:

Probable: Need to satisfy any two of the symptoms described in (i) and any of the epidemiological history described in (ii):

i. Clinical symptoms: (1) fever; (2) imaging showing pneumonia typical of the disease; (3) during early disease, total white cells normal or reduced, or lymph cell count reduced

ii. Epidemiologic history: (1) within 2 weeks of symptom onset, travel or resident history in Wuhan region or other places with sustained local transmission; (2) within 2 weeks of symptom onset, contact with persons from Wuhan city or other places with sustained local transmission who had fever with respiratory symptoms, (3) belong to a cluster or epidemiologic connection with COVID-19 infected persons.

Confirmed: Need to satisfy criteria for probable case and have a RT-qPCR positive result from respiratory or blood samples, or genome sequencing highly similar with known SARS-CoV-2. available strains from lab test of respiratory or blood samples.

Comparing predictive models of epidemic trajectories

To evaluate hypotheses regarding the effect of mobility and testing on COVID-19 dynamics, we fit three different Generalized Linear Models (GLM). Model 1 was a Poisson GLM to estimate daily case counts, Model 2 was a negative binomial GLM to estimate daily case counts, and Model 3 was a log-linear regression to estimate daily cumulative cases. BIC scores shown in Fig. 4b are calculated on a GLM of the form $Y(t) = Y(t-4) + IT(t) + M(t-5) + IM(t)$ where $Y(t)$ is either the number of new cases observed on day t (Model 1 & 2) or cumulative number of cases observed through day t (Model 3), $Y(t-4)$ represents the number of cases (or the cumulative number under Model 3) four days prior (median doubling time outside Hubei province), $IT(t)$ is an indicator function for RT-qPCR test availability that is 1 after 19th January 2020 and 0 before, $M(t-5)$ is the Baidu Inc-estimated mobility between Wuhan and each province 5 days prior (median incubation period), and $IM(t)$ is an indicator function which is set to 1 after 26th January 2020 and 0 before (which represents one median incubation period from 22nd January 2020). Models were fit to province-level data. The three models were compared using differences in Bayesian Information Criteria (BIC), where larger values indicate models with lower relative support, and BIC>4 considered the cutoff for substantial model improvement. We performed a detailed sensitivity analysis on the availability of RT-qPCR tests, doubling time, and incubation periods. We obtained qualitatively similar results for Model 1 (Poisson GLM fit to daily case counts), Model 2 (negative binomial GLM fit to daily case counts), and Model 3 (log-linear regressions fit to cumulative cases), see Table S2. In addition, we provide a full time series analysis of the optimal lag structure for cases and mobility for each province. Additionally, although BIC is considered more conservative, model selection results were confirmed using AIC for model selection (see Fig. 4 and Table S2). Lastly, we validated our model selection

results using elastic-net regression and n-fold cross validation as implemented in the R package GLMNET v. 2.0-18 (*35*, *36)*.

Estimating epidemic doubling time

To estimate the epidemic doubling time across each province, we fit a mixed effects Poisson GLM of daily case counts to days since the first case report in each province (fixed effect) and a random effect for each province on the slope and intercept, using the R package lme4 v.1.1-21 (*37*). Daily case counts were determined using the date of symptom onset. However, we only have a symptom onset date for 667 cases. Where the date of symptom onset was not available, we estimated the symptom onset date based on a linear regression model where symptom onset date was fit to confirmation date (n =632 with both onset and confirmation dates, $p < 0.001$, $R^2 = 0.77$). Using this model, we estimated the onset date for the 31,436 cases with a recorded confirmation date.

Model selection via elastic-net regression and cross-validation

We fit regularized Poisson and negative binomial models with an elastic net penalty, i.e., 50/50 mixture of the lasso and ridge penalties, with the regularization coefficient (lambda) selected by leave-one-out cross-validation. As seen with the AIC/BIC-based model selection, the regularized model included terms for lagged cases, the mobility and testing indicator variables, and mobility out of Wuhan. The out-of-sample log likelihood for the regularized Poisson regression was -9102 and was -22519 for the negative binomial model. The significantly worse fit for the negative binomial model was primarily driven by two outlier predictions, removing those results in an out-of-sample log likelihood for the negative binomial model of -11625. Because GLMNET has

not implemented a negative binomial model, we performed regularization using the Poisson model and estimated the overdispersion parameter using the glm.nb function in the R package MASS v. 7.3-51.4 (*37*). All code and data are available here (*38*).

Supplementary text

To ascertain whether earlier travel restrictions could have prevented the wide-spread increase in cases witnessed in late-January we constructed a simple forecasting model for COVID-19. Briefly, we forecast the cumulative number of cases in each Chinese province by simply doubling the number of cumulative cases reported six days prior. For dates prior to Jan. 28th and after Feb 3rd, this naive forecast produces an accurate estimate of the cumulative number of cases in each province **(Fig. S4)**. However, the cumulative number of cases reported on Jan 28th is poorly estimated using this model (**Fig. S4).** In order to accurately forecast the number of cases on Jan 28th, we must also include the relative amount of mobility out of Wuhan into various provinces in the regression model. In **Fig. S4**, we show how a model including only movement from Wuhan on January 22nd fit to the residuals from **Fig. S4** is once again able to accurately forecast cumulative cases. This indicates that for any hope of success of controlling the spread of an epidemic, movement restrictions must be prompt.

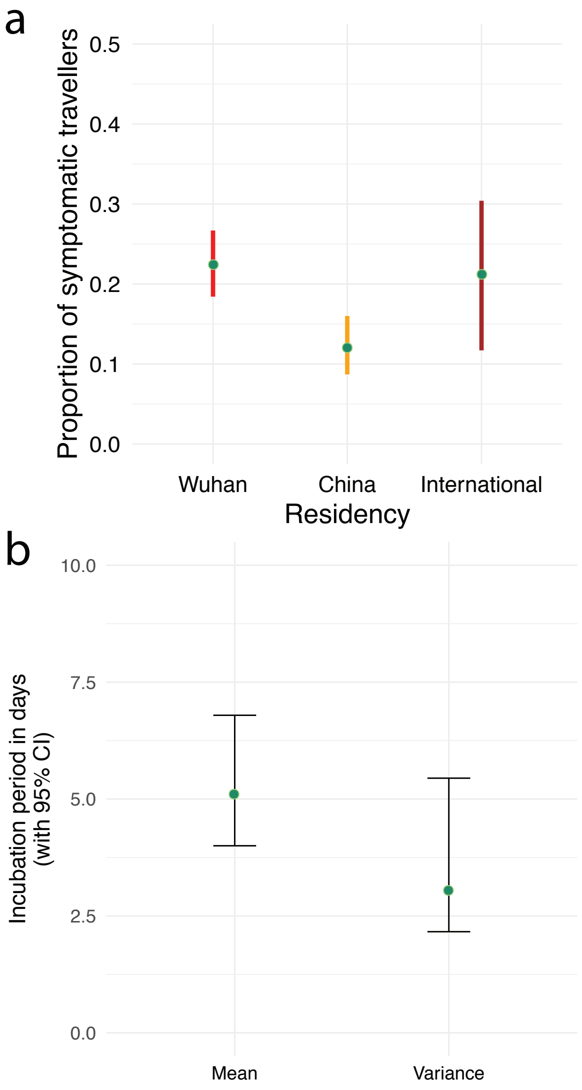

Fig. S1. a) Dates of symptom onset before date of travel from Wuhan. b) Incubation period estimates and standard deviation.

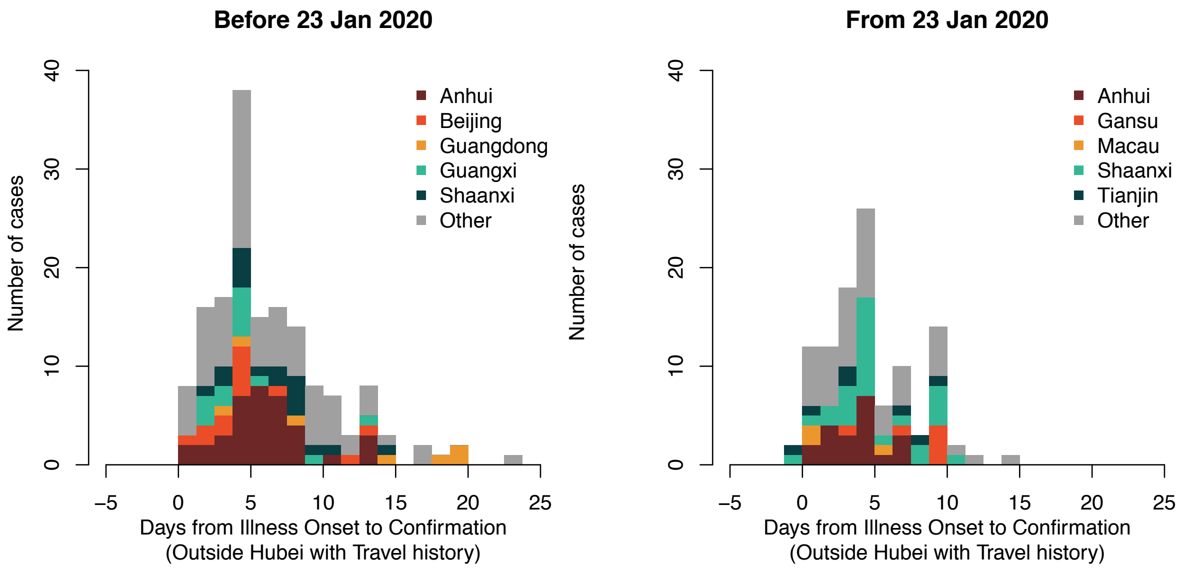

Fig. S2. Interval between symptom onset and date of confirmation in confirmed cases with reported travel history in two key periods, before and after January 23, 2020.

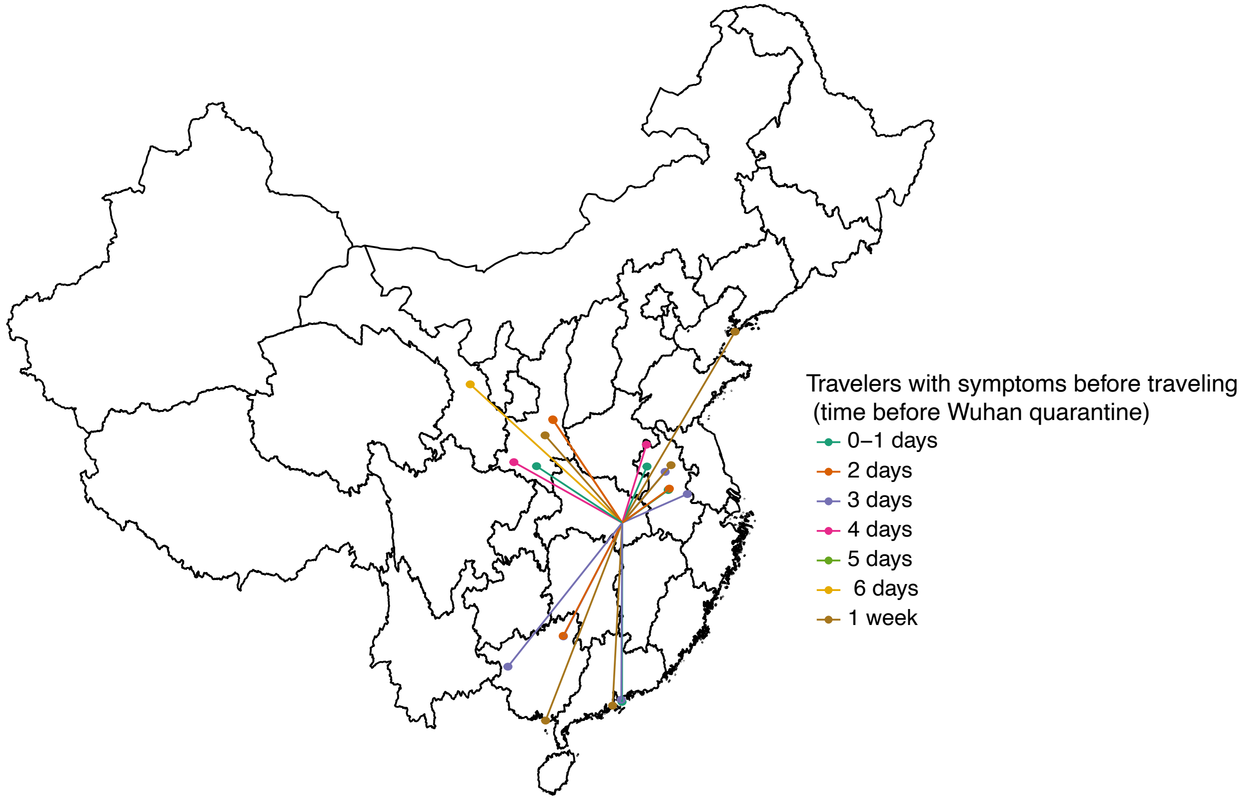

Fig. S3. Map of confirmed cases of COVID-19 with known travel history and date of onset date before date of travel.

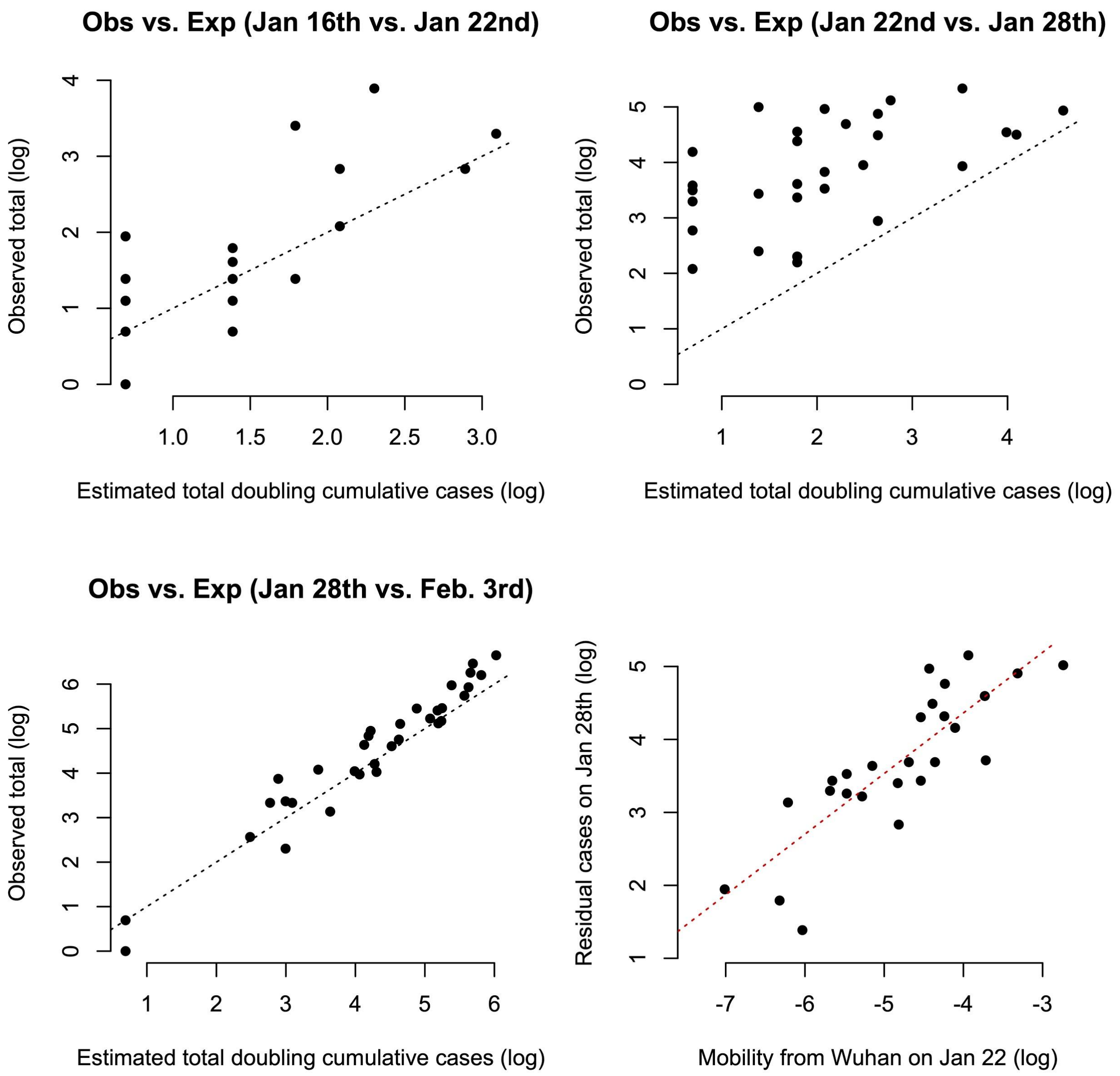

Fig. S4. Predicting COVID-19 cases using mobility data. a) Province-level cumulative cases on January 22nd can be accurately predicted based on simply doubling the number of cumulative number of cases occurring on January 16th. b) However, by Jan. 28th, the expected number of cases has significantly increased with respect to predictions based on cases through January 22nd. c) By Feb. 34rd, cumulative cases are once again well estimated based on the cumulative number of cases in each province six days earlier, i.e., on Jan. 28th. d) The deviation in cases on

January 28th is well explained by the relative amount of migration out of Wuhan on January 22nd.

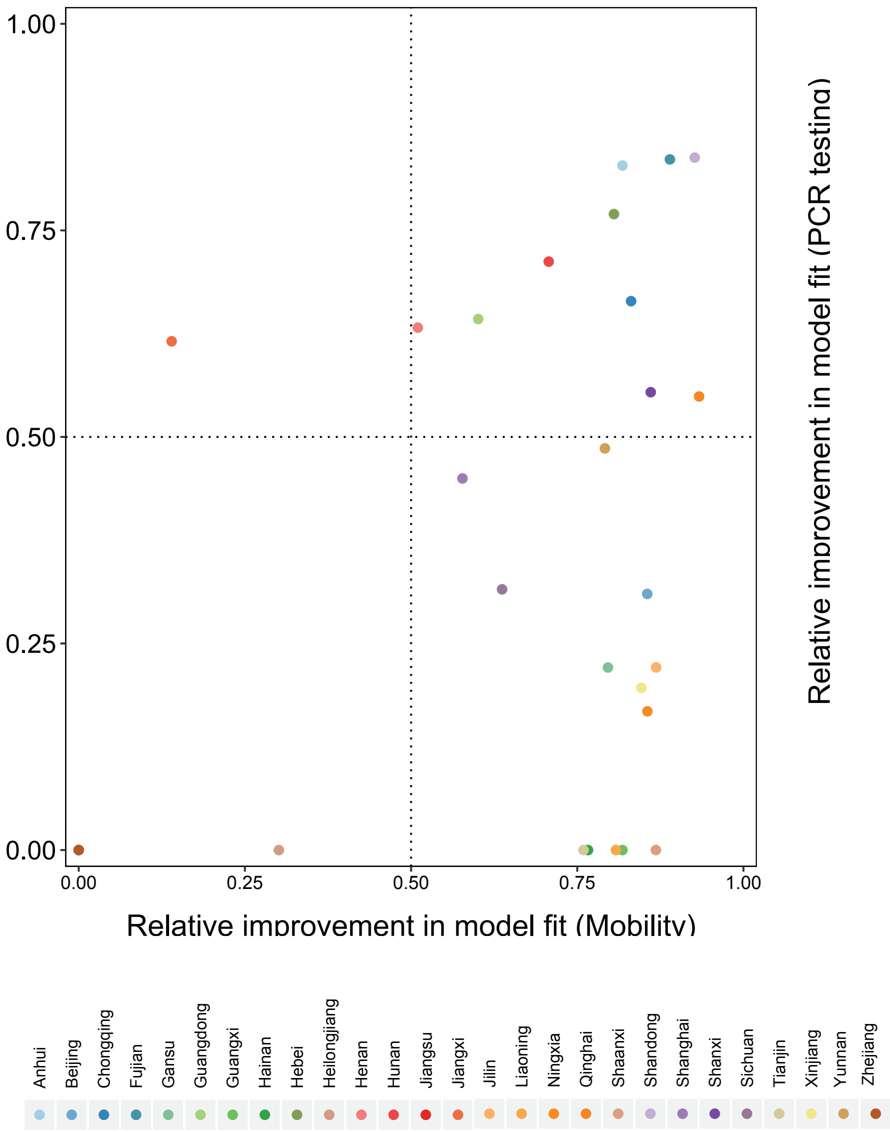

Fig. S5. Relative importance of RT-qPCR testing vs. human mobility to improve a simple GLM of COVID-19 when estimating exponential growth in province-level cases. Relative improvement is measured as one minus the residuals of a GLM with lagged cases + RT-qPCR testing availability (y-axis) and a GLM with lagged cases + mobility from Wuhan. Values were normalized by the observed number of cases such that they ranged between 0 and 1. The resulting metric has a value of 0 for a model where the residual error vastly eclipses the observed data and a value of 1 when residual error is 0, i.e., a perfect model fit.

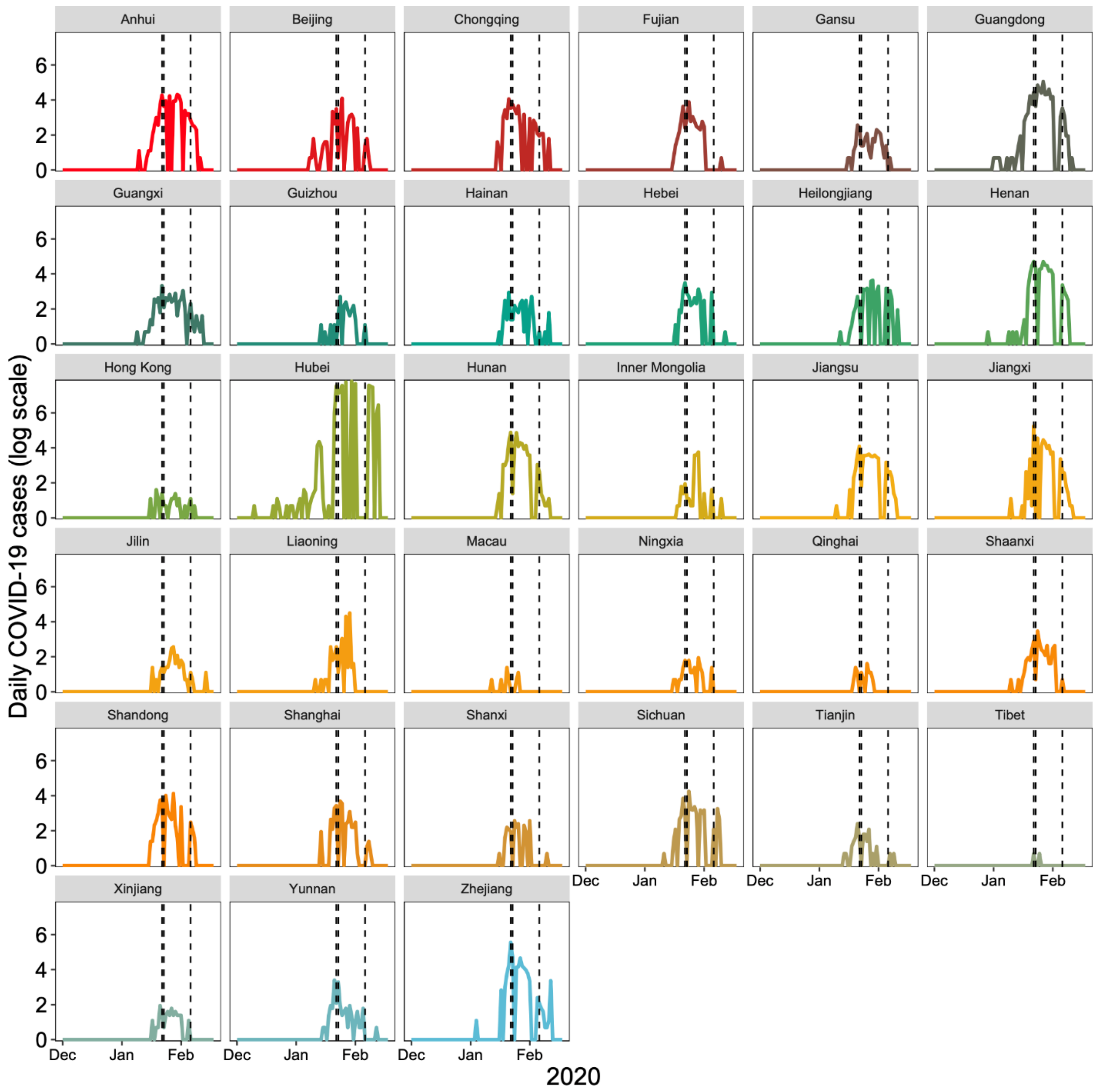

Fig. S6. Daily case counts of COVID-19 in China between January 1st and February 15th, 2020 (log scale).

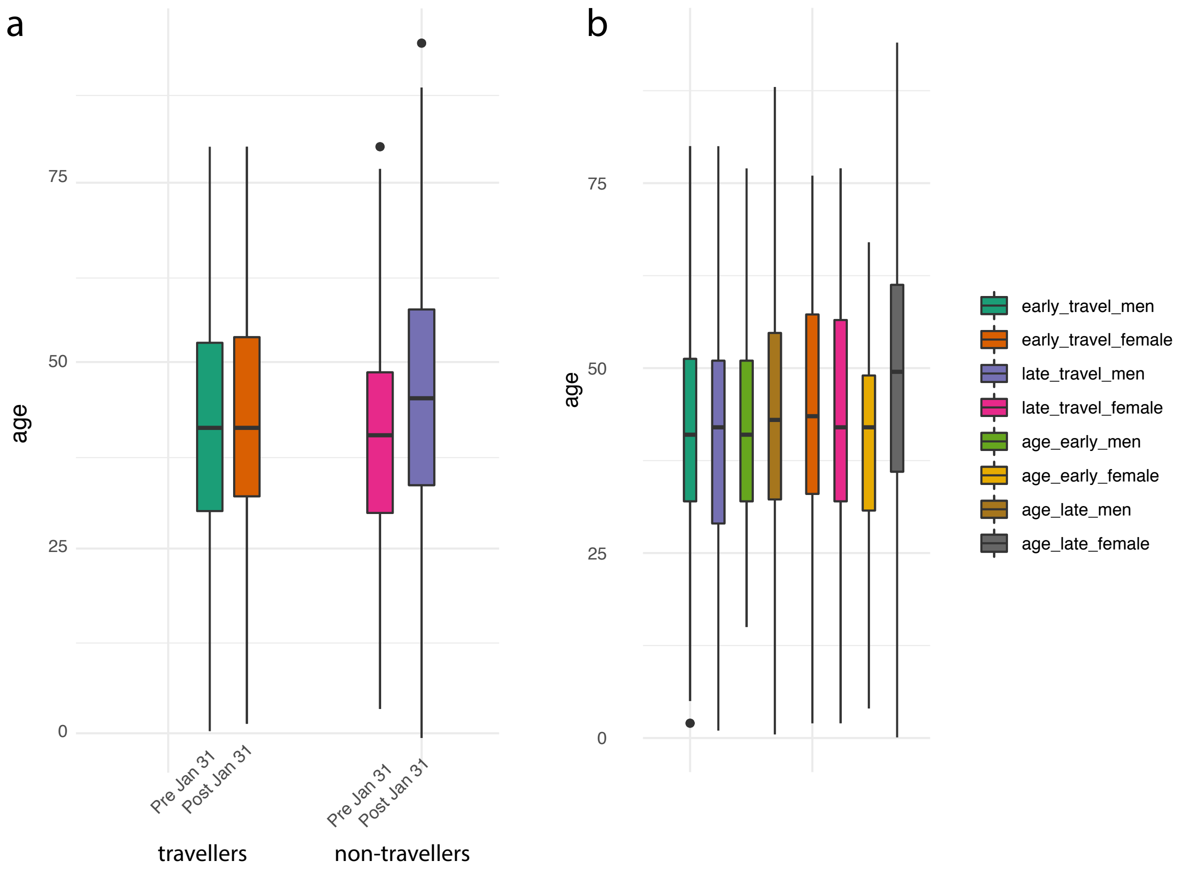

Fig. S7. a) Boxplots of data on age of cases reporting travel history and those that did not before 31 January 2020 and after. b) Boxplots of data on age and sex of cases reporting travel history and those that did not before 31 January 2020 and after. The box and whiskers show the median, interquartile range, and 95% credible intervals derived from the detailed line list data.

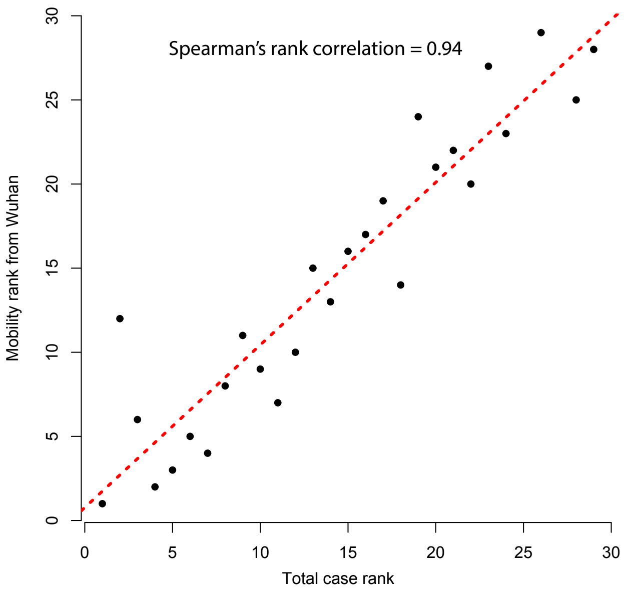

Fig. S8. Correlation between total number of cases and human mobility from Wuhan.

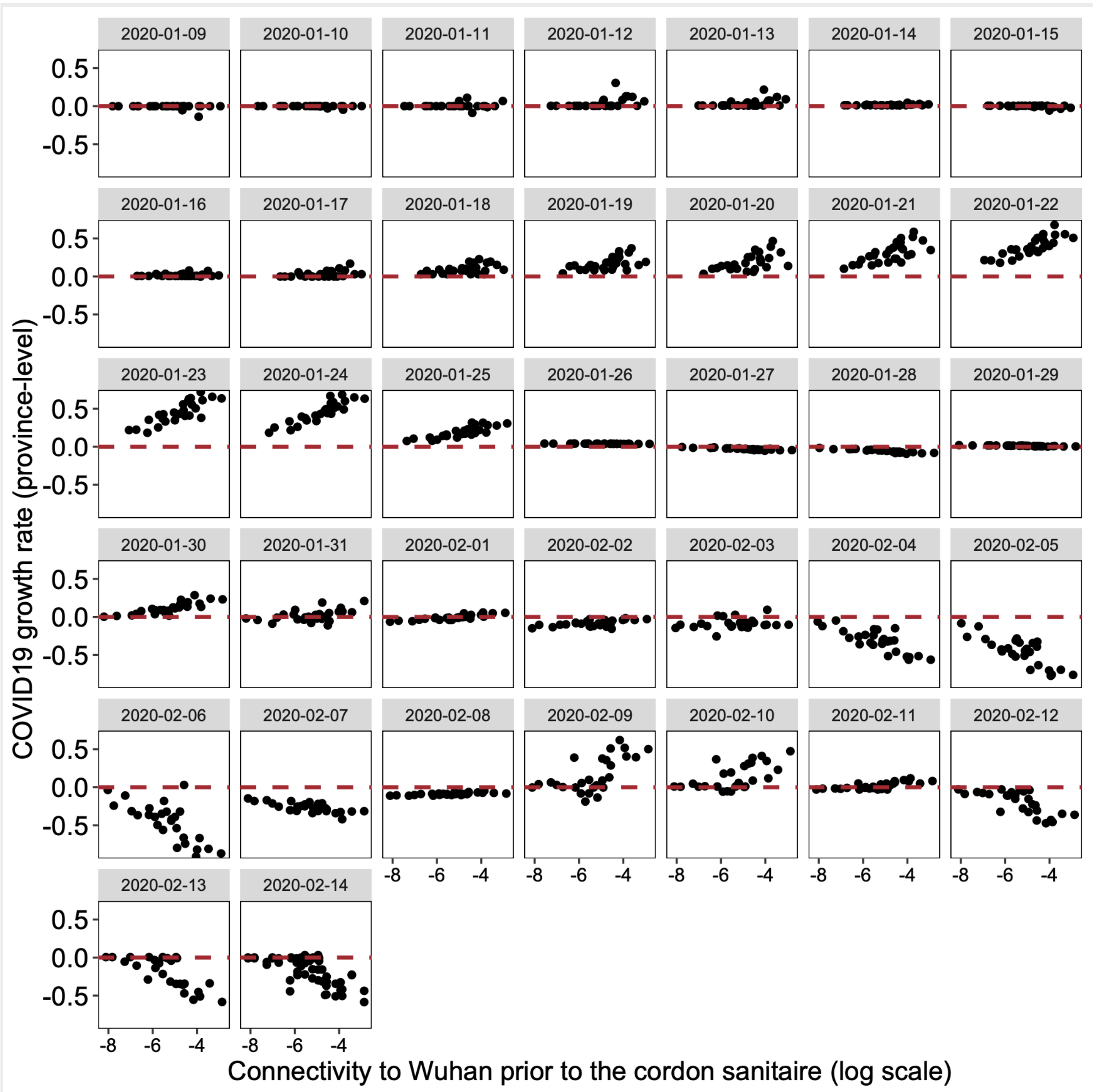

Fig. S9. Time series of province-level growth rates of the COVID-19 epidemic in provinces in China. Estimates of the growth rate were obtained by performing a time-series analysis using mixed-effect model of lagged, log linear daily case counts in each province to overlapping, rolling seven-day windows. Above the red line are positive growth rates and below are growth rates that are negative. Relationship between the growth rate and human mobility at different times of the epidemic. Blue indicates before the implementation of the cordon sanitaire and green after. Similarly, data are based on log linear daily case counts in each province to overlapping, rolling seven-day windows. Note that daily case data are subject to fluctuations in

testing.

Date	Poisson (pseudo R^2)	Negative Binomial (pseudo R^2)
01-14-2020	0.03	0.03
01-18-2020	0.09	0.10
01-25-2020	0.94	0.42
01-29-2020	0.99	0.70

Table S1. Table shows the pseudo- R^2 values for Poisson and Negative Binomial GLM of daily case counts and 5-day lagged log mobility from Wuhan, where pseudo- R^2 were calculated using model deviances as described (*39*, *40*).

Model	LM-AIC	Pois-AIC	NB-AIC	LM-BIC	Pois-BIC	NB-BIC
CASES_lag4	6339.09826	38354.086	6805.23754	6356.48767	38365.6789	6822.62694
CASES_lag4-TEST	6239.93575	28208.9046	6482.52214	6263.12162	28226.294	6505.70802
CASES_lag4-MOB	5538.26436	31090.6513	6134.41458	5560.77064	31107.531	6156.92086
CASES_lag4-MOB_IND	6310.48254	38191.6028	6807.21563	6333.66841	38208.9922	6830.4015
CASES_lag4-TEST-MOB	5405.28156	21068.2595	5729.17677	5433.41441	21090.7658	5757.30962
CASES_lag4-MOB_IND-MOB	5520.70815	31000.071	6133.96156	5548.841	31022.5773	6162.09442
CASES_lag4-MOB_IND-MOB-TEST	4971.61954	17807.4577	5676.43891	5005.37896	17835.5906	5710.19833

Table S2. Table shows the AIC and BIC values for a log-linear regression based on cumulative cases, a Poisson GLM of daily case counts and a Negative Binomial GLM of daily case counts using seven combinations of predictors.

Members of the Open COVID-19 Working Group:
Bo Xu, Sumiko Mekaru, Kara Sewalk, Lauren Goodwin, Alyssa Loskill, Emily Cohn, Yulin Hswen, Sarah C. Hill, Maria M. Cobo, Anastasia Lambrou, Isha Berry, Alexander Zarebski, Sabrina Li, Erin Hulland, Julia Morgan, Lin Wang, Katelynn O'Brien

References and Notes

1. S. Chen, J. Yang, W. Yang, C. Wang, T. Bärnighausen, COVID-19 control in China during mass population movements at New Year. *Lancet* **395**, 764–766 (2020). doi:10.1016/S0140-6736(20)30421-9 Medline

2. N. Zhu, D. Zhang, W. Wang, X. Li, B. Yang, J. Song, X. Zhao, B. Huang, W. Shi, R. Lu, P. Niu, F. Zhan, X. Ma, D. Wang, W. Xu, G. Wu, G. F. Gao, W. Tan; China Novel Coronavirus Investigating and Research Team, A novel coronavirus from patients with pneumonia in China, 2019. *N. Engl. J. Med.* **382**, 727–733 (2020). doi:10.1056/NEJMoa2001017 Medline

3. H. Tian, Y. Liu, Y. Li, C.-H. Wu, B. Chen, M. U. G. Kraemer, B. Li, J. Cai, B. Xu, Q. Yang, B. Wang, P. Yang, Y. Cui, Y. Song, P. Zheng, Q. Wang, O. N. Bjornstad, R. Yang, B. Grenfell, O. Pybus, C. Dye, The impact of transmission control measures during the first 50 days of the COVID-19 epidemic in China. medRxiv 2020.01.30.20019844 [Preprint]. 10 March 2020; https://doi.org/10.1101/2020.01.30.20019844.

4. Z. Du, L. Wang, S. Cauchemez, X. Xu, X. Wang, B. J. Cowling, L. A. Meyers, Risk for transportation of 2019 novel coronavirus disease from Wuhan to other cities in China. *Emerg. Infect. Dis.* **26** (2020). doi:10.3201/eid2605.200146 Medline

5. J. T. Wu, K. Leung, G. M. Leung, Nowcasting and forecasting the potential domestic and international spread of the 2019-nCoV outbreak originating in Wuhan, China: A modelling study. *Lancet* **395**, 689–697 (2020). doi:10.1016/S0140-6736(20)30260-9 Medline

6. S. Zhao, Z. Zhuang, P. Cao, J. Ran, D. Gao, Y. Lou, L. Yang, Y. Cai, W. Wang, D. He, M. H. Wang, Quantifying the association between domestic travel and the exportation of novel coronavirus (2019-nCoV) cases from Wuhan, China in 2020: A correlational analysis. *J. Travel Med.* 10.1093/jtm/taaa022 (2020). doi:10.1093/jtm/taaa022 Medline

7. World Health Organization (WHO), *Coronavirus Disease 2019 (COVID-19) Situation Report – 27* (2020); https://www.who.int/docs/default-source/coronaviruse/situation-reports/20200216-sitrep-27-covid-19.pdf?sfvrsn=78c0eb78_2.

8. B. Xu, B. Gutierrez, S. Mekaru, K. Sewalk, L. Goodwin, A. Loskill, E. L. Cohn, Y. Hswen, S. C. Hill, M. M. Cobo, A. E. Zarebski, S. Li, C.-H. Wu, E. Hulland, J. D. Morgan, L. Wang, K. O'Brien, S. V. Scarpino, J. S. Brownstein, O. G. Pybus, D. M. Pigott, M. U. G. Kraemer, Epidemiological data from the COVID-19 outbreak, real-time case information. *Sci. Data* **7**, 106 (2020). doi:10.1038/s41597-020-0448-0

9. S. Cauchemez, C. Fraser, M. D. Van Kerkhove, C. A. Donnelly, S. Riley, A. Rambaut, V. Enouf, S. van der Werf, N. M. Ferguson, Middle East respiratory syndrome coronavirus: Quantification of the extent of the epidemic, surveillance biases, and transmissibility. *Lancet Infect. Dis.* **14**, 50–56 (2014). doi:10.1016/S1473-3099(13)70304-9 Medline

10. J. Lessler, N. G. Reich, R. Brookmeyer, T. M. Perl, K. E. Nelson, D. A. T. Cummings, Incubation periods of acute respiratory viral infections: A systematic review. *Lancet Infect. Dis.* **9**, 291–300 (2009). doi:10.1016/S1473-3099(09)70069-6 Medline

11. J. A. Backer, D. Klinkenberg, J. Wallinga, Incubation period of 2019 novel coronavirus

(2019-nCoV) infections among travellers from Wuhan, China, 20-28 January 2020. *Euro Surveill.* **25**, 20–28 (2020). doi:10.2807/1560-7917.ES.2020.25.5.2000062 Medline

12. Q. Li, X. Guan, P. Wu, X. Wang, L. Zhou, Y. Tong, R. Ren, K. S. M. Leung, E. H. Y. Lau, J. Y. Wong, X. Xing, N. Xiang, Y. Wu, C. Li, Q. Chen, D. Li, T. Liu, J. Zhao, M. Li, W. Tu, C. Chen, L. Jin, R. Yang, Q. Wang, S. Zhou, R. Wang, H. Liu, Y. Luo, Y. Liu, G. Shao, H. Li, Z. Tao, Y. Yang, Z. Deng, B. Liu, Z. Ma, Y. Zhang, G. Shi, T. T. Y. Lam, J. T. K. Wu, G. F. Gao, B. J. Cowling, B. Yang, G. M. Leung, Z. Feng, Early transmission dynamics in Wuhan, China, of novel coronavirus–infected pneumonia. *N. Engl. J. Med.* NEJMoa2001316 (2020). doi:10.1056/NEJMoa2001316 Medline
13. T. J. Hastie, D. Pregibon, "Generalized linear models" in *Statistical Models in S*, J. M. Chambers, T. J. Hastie, Eds. (Wadsworth & Brooks/Cole, 1992), pp. 195–246.
14. J. Riou, C. L. Althaus, Pattern of early human-to-human transmission of Wuhan 2019 novel coronavirus (2019-nCoV), December 2019 to January 2020. *Euro Surveill.* **25**, 1–5 (2020). doi:10.2807/1560-7917.ES.2020.25.4.2000058 Medline
15. A. R. Tuite, D. N. Fisman, Reporting, epidemic growth, and reproduction numbers for the 2019 novel coronavirus (2019-nCoV) epidemic. *Ann. Intern. Med.* (2020). doi:10.7326/M20-0358 Medline
16. M. J. Keeling, O. N. Bjørnstad, B. T. Grenfell, "Metapopulation dynamics of infectious diseases" in *Ecology, Genetics and Evolution of Metapopulations*, I. Hanski, O. E. Gaggiotti, Eds. (Elsevier, 2004), pp. 415–445.
17. D. J. Watts, R. Muhamad, D. C. Medina, P. S. Dodds, Multiscale, resurgent epidemics in a hierarchical metapopulation model. *Proc. Natl. Acad. Sci. U.S.A.* **102**, 11157–11162 (2005). doi:10.1073/pnas.0501226102 Medline
18. World Health Organization, *Report of the WHO-China Joint Mission on Coronavirus Disease 2019 (COVID-19)* (WHO, 2020); https://www.who.int/docs/default-source/coronaviruse/who-china-joint-mission-on-covid-19-final-report.pdf.
19. Z. Wu, J. M. McGoogan, Characteristics of and important lessons from the coronavirus disease, 2019 (covid-19) outbreak in china: summary of a Report of 72,314 cases from the Chinese Center for Disease Control and Prevention.. *JAMA* **2019**, 17–20 (2020). doi:10.1001/jama.2020.2648 Medline
20. K. P. Burnham, D. R. Anderson, Multimodel inference. *Sociol. Methods Res.* **33**, 261–304 (2004). doi:10.1177/0049124104268644
21. Novel Coronavirus Pneumonia Emergency Response Epidemiology Team, The epidemiological characteristics of an outbreak of 2019 novel coronavirus diseases (COVID-19) in China [in Chinese]. *Zhonghua Liu Xing Bing Xue Za Zhi* **41**, 145–151 (2020). Medline
22. E. Goldstein, V. E. Pitzer, J. J. O'Hagan, M. Lipsitch, Temporally varying relative risks for infectious diseases: Implications for infectious disease control.. *Epidemiology* **28**, 136–144 (2017). doi:10.1097/EDE.0000000000000571 Medline
23. C. Fraser, S. Riley, R. M. Anderson, N. M. Ferguson, Factors that make an infectious disease outbreak controllable. *Proc. Natl. Acad. Sci. U.S.A.* **101**, 6146–6151 (2004).

doi:10.1073/pnas.0307506101 Medline

24. R. Li, S. Pei, B. Chen, Y. Song, T. Zhang, W. Yang, J. Shaman, Substantial undocumented infection facilitates the rapid dissemination of novel coronavirus (COVID-19). medRxiv 2020.02.14.20023127 [Preprint]. 17 February 2020; https://doi.org/10.1101/2020.02.14.20023127.

25. M. U. G. Kraemer, C.-H. Yang, B. Gutierrez, C.-H. Wu, B. Klein, D. Pigott, Open COVID-19 Data Working Group, L. du Plessis, N. R. Faria, R. Li, W. P. Hanage, J. S. Brownstein, M. Layan, A. Vespignani, H. Tian, C. Dye, O. G. Pybus, S. V. Scarpino, Code for: The effect of human mobility and control measures on the COVID-19 epidemic in China. Zenodo (2020); https://doi.org/10.5281/zenodo.3714914.

26. B. Xu, M. U. G. Kraemer; Open COVID-19 Data Curation Group, Open access epidemiological data from the COVID-19 outbreak. *Lancet Infect. Dis.* **3099**, 30119 (2020). doi:10.1016/S1473-3099(20)30119-5 Medline

27. R. E. Ramshaw, I. D. Letourneau, A. Y. Hong, J. Hon, J. D. Morgan, J. C. P. Osborne, S. Shirude, M. D. Van Kerkhove, S. I. Hay, D. M. Pigott, A database of geopositioned Middle East Respiratory Syndrome Coronavirus occurrences. *Sci. Data* **6**, 318 (2019). doi:10.1038/s41597-019-0330-0 Medline

28. M. L. McHugh, The chi-square test of independence. *Biochem. Med. (Zagreb)* **23**, 143–149 (2013). doi:10.11613/BM.2013.018 Medline

29. J. H. McDonald, *Handbook of Biological Statistics* (Sparky House, ed. 3, 2014).

30. N. R. Faria, M. U. G. Kraemer, S. C. Hill, J. Goes de Jesus, R. S. Aguiar, F. C. M. Iani, J. Xavier, J. Quick, L. du Plessis, S. Dellicour, J. Thézé, R. D. O. Carvalho, G. Baele, C.-H. Wu, P. P. Silveira, M. B. Arruda, M. A. Pereira, G. C. Pereira, J. Lourenço, U. Obolski, L. Abade, T. I. Vasylyeva, M. Giovanetti, D. Yi, D. J. Weiss, G. R. W. Wint, F. M. Shearer, S. Funk, B. Nikolay, V. Fonseca, T. E. R. Adelino, M. A. A. Oliveira, M. V. F. Silva, L. Sacchetto, P. O. Figueiredo, I. M. Rezende, E. M. Mello, R. F. C. Said, D. A. Santos, M. L. Ferraz, M. G. Brito, L. F. Santana, M. T. Menezes, R. M. Brindeiro, A. Tanuri, F. C. P. Dos Santos, M. S. Cunha, J. S. Nogueira, I. M. Rocco, A. C. da Costa, S. C. V. Komninakis, V. Azevedo, A. O. Chieppe, E. S. M. Araujo, M. C. L. Mendonça, C. C. Dos Santos, C. D. Dos Santos, A. M. Mares-Guia, R. M. R. Nogueira, P. C. Sequeira, R. G. Abreu, M. H. O. Garcia, A. L. Abreu, O. Okumoto, E. G. Kroon, C. F. C. de Albuquerque, K. Lewandowski, S. T. Pullan, M. Carroll, T. de Oliveira, E. C. Sabino, R. P. Souza, M. A. Suchard, P. Lemey, G. S. Trindade, B. P. Drumond, A. M. B. Filippis, N. J. Loman, S. Cauchemez, L. C. J. Alcantara, O. G. Pybus, Genomic and epidemiological monitoring of yellow fever virus transmission potential. *Science* **361**, 894–899 (2018). doi:10.1126/science.aat7115 Medline

31. M. U. G. Kraemer, R. C. Reiner Jr., O. J. Brady, J. P. Messina, M. Gilbert, D. M. Pigott, D. Yi, K. Johnson, L. Earl, L. B. Marczak, S. Shirude, N. Davis Weaver, D. Bisanzio, T. A. Perkins, S. Lai, X. Lu, P. Jones, G. E. Coelho, R. G. Carvalho, W. Van Bortel, C. Marsboom, G. Hendrickx, F. Schaffner, C. G. Moore, H. H. Nax, L. Bengtsson, E. Wetter, A. J. Tatem, J. S. Brownstein, D. L. Smith, L. Lambrechts, S. Cauchemez, C. Linard, N. R. Faria, O. G. Pybus, T. W. Scott, Q. Liu, H. Yu, G. R. W. Wint, S. I. Hay, N. Golding, Past and future spread of the arbovirus vectors Aedes aegypti and Aedes

albopictus. *Nat. Microbiol.* **4**, 854–863 (2019). doi:10.1038/s41564-019-0376-y Medline

32. G. Jin, J. Yu, L. Han, S. Duan, The impact of traffic isolation in Wuhan on the spread of 2019-nCov. medRxiv 2020.02.04.20020438 [Preprint]. 5 February 2020; https://doi.org/10.1101/2020.02.04.20020438.

33. S. Ai, G. Zhu, F. Tian, H. Li, Y. Gao, Y. Wu, Q. Liu, H. Lin, Population movement, city closure and spatial transmission of the 2019-nCoV infection in China. medRxiv 2020.02.04.20020339 [Preprint]. 5 February 2020; https://doi.org/10.1101/2020.02.04.20020339.

34. Aurora Big Data, *2017 Mobile Map App Research Report: Which of the Highest, the Baidu, and Tencent Is Strong? [in Chinese]* (2017); https://baijiahao.baidu.com/s?id=1590386747028939917&wfr=spider&for=pc.

35. D. Bates, M. Mächler, B. Bolker, S. Walker, Fitting linear mixed-effects models using lme4. *J. Stat. Softw.* **67**, 201–210 (2015). doi:10.18637/jss.v067.i01

36. J. Friedman, T. Hastie, R. Tibshirani, Regularization paths for generalized linear models via coordinate descent. *J. Stat. Softw.* **33**, 1–22 (2010). doi:10.18637/jss.v033.i01 Medline

37. B. Ripley, B. Venables, D. M. Bates, K. Hornik, A. Gebhardt, D. Firth, MASS (R Package Version 7.3-51.5, 2019); https://cran.r-project.org/package=MASS.

38. M. U. G. Kraemer, C.-H. Yang, B. Gutierrez, C.-H. Wu, B. Klein, D. M. Pigott, Open COVID-19 Data Working Group, L. du Plessis, N. R. Faria, R. Li, W. P. Hanage, J. S. Brownstein, M. Layan, A. Vespignani, H. Tian, C. Dye, O. G. Pybus, S. V. Scarpino, Code for: The effect of human mobility and control measures on the COVID-19 epidemic in China, Zenodo (2020); https://doi.org/10.5281/zenodo.3714914.

39. H. Heinzl, M. Mittlböck, Pseudo R-squared measures for Poisson regression models with over- or underdispersion. *Comput. Stat. Data Anal.* **44**, 253–271 (2003). doi:10.1016/S0167-9473(03)00062-8

40. A. Zeileis, C. Kleiber, S. Jackman, Regression models for count data in R. *J. Stat. Softw.* **27**, 1–25 (2008). doi:10.18637/jss.v027.i08
